# An Improved Design and Implementation of a Range-Controlled Communication System for Mobile Phones

**DOI:** 10.3390/s20174997

**Published:** 2020-09-03

**Authors:** Mingyang Gong, Haichun Zhang, Zhenglin Liu

**Affiliations:** School of Optical and Electronic Information, Huazhong University of Science and Technology, Wuhan 430074, China; d201677550@hust.edu.cn (M.G.); d201880640@hust.edu.cn (H.Z.)

**Keywords:** range-controlled communication, low-frequency circuits, rail-to-rail amplifier

## Abstract

The Short-range-controlled communication system (RCC) based on a subscriber identity module (SIM) card is a replacement for the standard near-field communication (NFC) system to support near-field payment applications. The RCC uses both the low-frequency (LF) and high-frequency (HF) wireless communication system. The RCC communication distance is controlled under 10 cm. However, current RCCs suffer from compatibility issues, and the LF communication distance is lower than 0.5 cm in some phones with completely metallic shells. In this paper, we propose an improved LF communication system design, including an LF transmitter circuit, LF receiver chip, and LF-HF communication protocol. The LF receiver chip has a rail-to-rail amplifier and a self-correcting clock recovery differential Manchester decoder, which do not have the limitations of accurate gain and high system clock. The LF receiver chip is fabricated in a 0.18 μm CMOS technology platform, with a die size of 1.05 mm × 0.9 mm and current consumption of 41 μA. The experiments show that the improved RCC has better compatibility, and the communication distance reaches to 4.2 cm in phones with completely metallic shells.

## 1. Introduction

Payment applications based on smartphones change peoples’ daily lives and make the world a better place. Two-dimensional barcode payment systems and face recognition payment systems utilize the cameras and powerful resources of smartphones, and they also require an advanced mobile network infrastructure, which is not always available in underdeveloped countries. The standard near-field communication (NFC) system is based on the International Organization for Standardization (ISO) protocol 14443 at 13.56 MHz [1,2,3,4]. It can be used for both payment and for access control and other applications [5,6,7,8]. NFC does not require a mobile network and is especially suitable for underdeveloped countries with imperfect mobile network infrastructure [9]. NFC wireless communication needs a reader tuning coil and a receiver tuning coil. If the coupling tuning coil in phones is too small, then the signal from the phones to the NFC reader will be weak, and communication may fail [10]. The shielding of the phone’s mainboard and metallic shells make the situation even worse when placing the coil in a subscriber identity module (SIM) card [11]. The coil is always placed on the back cover of the smartphone [12,13], with a 13.56 MHz NFC transceiver chip in the mainboard.

Many people are still using cheap feature phones, especially in underdeveloped countries. These feature phones have neither powerful resources for image recognition nor 13.56 MHz NFC. Fortunately, external component SIM cards can be used to support inserted wireless communication [14]. SIM cards contain a secure element (SE) chip with the same security level as a bank card and usually meet the Common Criteria evaluation assurance level (EAL) 4+ standard. The SE chip communicates with the mobile phone using the ISO7816 protocol or single wire protocol (SWP). Some works have integrated a high-frequency (HF) radio chip into a SIM card (RF-SIM) to implement specific applications [15,16]. The HF chip is usually a 2.45 GHz, 433 MHz or sub-1 GHz wireless chip. Because the HF communication distance is at the meters level, this kind of RF-SIM card is usually used in garage door controls, indoor location tracking, and physical access control applications [17,18,19]. The HF communication distance may be as long as 100 m and is not suitable for payment applications because there may be many people in the range of wireless communication waiting to pay.

Decreasing the transmission power of HF is a way to restrict the HF communication distance. As a result of the different manufacturing materials of phone shells and the SIM card’s location in the phone, each phone needs to calibrate the HF transmission power after inserting an RF-SIM card. This kind of calibration needs professional tools, and the work involved is unfeasible. The Range-controlled communication (RCC) system is proposed to constraint the HF communication distance by adding a short-range low-frequency (LF) wireless chip. HF communication is started only when LF is activated, and the communication distance is constrained under 10 cm. Our previous work [20] presents a design for an LF receiver chip with a die size of 1850 μm by 1650 μm and a working current of 330 μA; it has a communication distance of 7.8 cm in phones with a non-metallic shell. However, some new mobile phones were produced in recent years that only accept the smallest nano-card. Some phones have a completely metallic shell and have a SIM card site which accepts up to three cards. The inner card of SIM card site has weakened LF signals, and the metal shell makes it even worse. The SIM card’s working distance based on the LF receiver chip in [20] decreases to less than 0.5 cm in these new mobile phones. The large die size makes it difficult to increase LF coil turn number in nano-SIM cards. Besides, to obtain a better LF communication distance, all amplifiers in [20] should set to the largest gain, and the working current is much larger than 330 μA. Although it has an idle mode with 45 μA current consumption, in RCC application, the LF receiver is used to wakeup HF, and so is in working mode all the time.

In this paper, we propose an improved range-controlled communication system withan improved LF transmitter circuit, LF receiver chip, and LF-HF communication protocols. The major contributions of this work are: (1) the proposal of an improved RCC design which is even compatible with mobile phones with completely metallic shells. The die size of the LF receiver chip is decreased to 1.05 mm × 0.9 mm using a new analog frontend circuit, and more space is available for increasing the LF turning coil number in the SIM card. The proposed self-correcting clock recovery differential Manchester decoder and asynchronous serial peripheral interface (SPI) controller allows the LF receiver’s system clock to be as low as 50KHz, and the working current decreases to 41 μA; (2) the proposal of a low-frequency (LF) system with a transmitter and receiver chip, which is adapted from 2 kHz to 4 kHz without limiting the precise working frequency channel, makes the implementation cost of LF system much lower.

The remainder of this paper is organized as follows. Section 2 describes the system blocks of the range controlled communication system and discusses the LF communication model based on electromagnetic induction. Section 3 describes the LF transmitter circuit. Section 4 describes the LF receiver chip. Section 5 describes the communication protocols with HF and LF. The experimental setup and results are discussed in Section 6. Section 7 concludes the paper.

## 2. System Building Blocks

**Figure 1:**
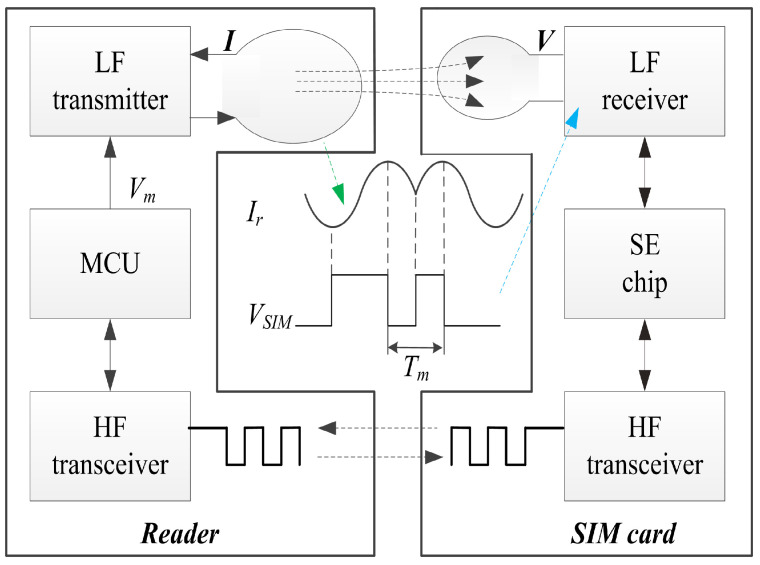
Building blocks of range-controlled communication system (RCC).

The RCC uses short-range LF communication to restrict the HF communication distance to [0, 10 cm] with proposed LF-HF protocols. Figure 1 shows the building blocks of the RCC, where communication is occurring between two devices: the master device with a reader circuit board and the slave device with an improved RF-SIM card. The reader circuit board consists of a microcontroller unit (MCU) that is responsible for communication, an HF transceiver, and an LF transmitter. The slave device includes an SE chip, an HF transceiver, and an LF receiver.

The LF circuit operates in the simplex mode, the message is transformed from the LF transmitter in the master device to LF receiver in the slave device, and there is no power transmission in the LF system. Assuming the tuning coil of LF transmitter is circular with a radius *r*, turn number $N_{r}$, and changing current $I_{r}$ for sending information. The changing current creates a magnetic field. The coupling coil in the SIM card is irregularly shaped, with turn number $N_{s}$ and area *S*. Assuming that two coils of the transmitter and receiver have an interval of *x*. The voltage $V_{s}$ induced in the receiver coil is as follows [20]:

(1) $$V_{s}\approx N_{r}N_{s}\mu_{0}Sr^{2}/\left(2\sqrt{{\left(r^{2}+x^{2}\right)}^{3}}\right)dI_{r}/dt$$

$\mu_{0}$ in (1) is the magnetic permeability of air or vacuum with a value of $4\pi \times 10^{-7}$. Equation (1) is applicable for two coils in the air. Actually, the two coils are not totally parallel. There is shell material between the two coils, which also affect the $V_{s}$:

(2) $$V_{s}\approx e^{-t\sqrt{\pi f\mu \gamma }}Qcos\alpha N_{r}N_{s}\mu_{0}Sr^{2}/\left(2\sqrt{{\left(r^{2}+x^{2}\right)}^{3}}\right)dI_{r}/dt$$

In Equation (2), *Q* is the quality factor of the resonant circuit; $e^{-t\sqrt{\pi f\mu \gamma }}$ is the magnetic-field attenuation factor [15]; *t* denote the thickness; *f* denotes the working frequency of LF; μ denotes the permeability; γ denotes the conductivity of the shielding material; and α is the angle between the transmitter coil and the coupling coil, when the two coils are parallel, α = 0.

LF uses magnetic coupling modulation to transform the differential Manchester code at the cm level. A large *x* signifies better implement of RCC reader equipment and user experience. The RCC is proposed to replace the NFC. Because the LF transmitter coil is always put in the place of the NFC reader coil in the reader equipment, so *r* and $N_{r}$ are constrained. The SIM card size is small, the micro-SIM card is 12 mm × 15 mm, the nano-SIM card has a smaller size of 12 mm × 9 mm. If the die size of the LF receiver chip is small, the LF receiver chip can have a package-in-package (PIP) bonding design with the SE chip, which can save some space to increase the tuning coil number. The die size of the LF receiver in previous work [20] was too large to perform PIP bonding with the SE chip. One method to obtain a larger *x* is to obtain a higher $V_{s}$; another method is to decrease the signal loss in analog frontend circuit of the LF receiver.

## 3. Low-Frequency Transmitter

**Figure 2:**
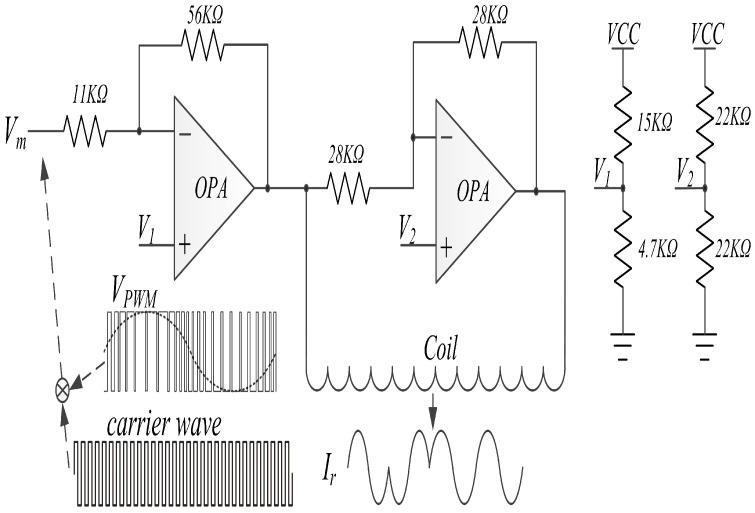
Low-frequency (LF) transmitter circuits.

The transmitting LF signal is modulated in the MCU as $V_{m}$ and sent to the LF transmitter circuits in the master device, as shown in Figure 1. From Equation (1), a large changing current is needed to generate a powerful magnetic field so that the LF receiver can function effectively. We use separate components to produce a large changing current, as shown in Figure 2. Two class-D amplifier chips are used to convert voltage $V_{m}$ into current $I_{r}$ at the LF coil and deliver wireless messages to the LF receiver. $V_{1}$ and $V_{2}$ are reference voltages without a precision requirement, and a simple resistance voltage divider circuit is used to generate $V_{1}$ and $V_{2}$.

$V_{m}$ is obtained using the pulse width modulation (PWM) method; the PWM is widely used to produce a sine-wave current for motor driver applications. The transmitting LF signal is a differential Manchester code, and the modulation is in the PWM mode, as shown by $V_{PWM}$ in Figure 2. A carrier waveform is added to increase the changes in $I_{r}$. The frequency of carrier waveform is usually at the MHz level and determined by the MCU.

## 4. Low Frequency Receiver

**Figure 3:**
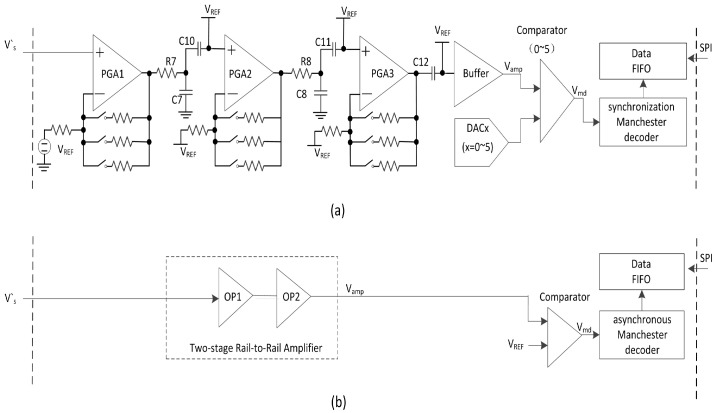
Analog frontend circuit: (**a**) in [20], (**b**) in this paper.

Limited by the size of the SIM card, the LF receiver is designed in a single chip with an LF analog frontend and digital logic. The analog frontend circuit is used to process the induced weak $V_{s}$ to the ideal digital signal $V_{SIM}$ in Figure 1. The SIM card, based on [20], has a high fail communication rate in phones with a completely metal shell, because the distance of LF communication is lower than 0.5 cm. The induced weak $V_{s}$ goes through three programmable gain amplifiers (PGA), a buffer is used to obtain large loading capacities, and six digital-to-analog convertors (DAC) and six comparators are used to convert the analog signal to a digital differential Manchester code and obtain the magnetic field intensity, as shown in Figure 3a. The magnetic-field intensity is used to judge whether the SIM card is in or out of the magnetic field. The signal processing path is too long, and there may be some signal loss or noise interference in these long processing paths. In this paper, we propose an improved analog frontend for LF receiver as shown in Figure 3b, a two-stage high gain rail-to-rail amplifier is used to amplify $V_{s}$, and a comparator is used to obtain $V_{SIM}$. The “in or out” decision of the magnetic field is made using digital logic.

### 4.1. Antenna Matching Circuits

**Figure 4:**
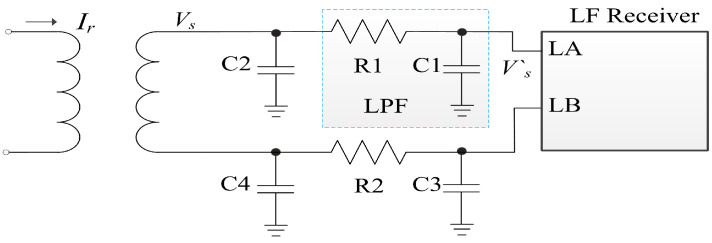
Matching circuits in the LF antenna coil.

Before the $V_{s}$ goes the rail-to-rail amplifiers, a matching circuit is inserted, as shown in Figure 4. In [20], R1, C1, C2, and R2, C3, C4 comprise two Π match networks. This kind of match network is usually used in NFC impedance matching of antenna coils to obtain the maximum communication distance [21,22,23]. The induced voltage in the 13.56 MHz NFC system is as high as the *V* level. The induced voltage $V_{s}$ in this proposed LF system is as low as the μV level. A Π matching network consumes the induced power and makes $V_{s}$ even worse.

Because the matching circuits are printed on SIM card substrate, we also bonded some SIM card from [20] with different values of resistance and capacitance, as shown in Figure 4. One removes R2, C3 and C4, and the other removes R2, C3, C4, and C2. The experiment shows the distance *x* is improved in both of the two new methods. The best is that with R1 and C1. Some phones with complitely metallic shells with this kind of SIM card can have a 0.2 ∼ 0.4 cm improvement. $R_{1}$ and $C_{1}$ comprise a lowpass filter (LPF), and the cut-off frequency $f_{c}$ = 1/(2π$R_{1}$$C_{1}$).

The LF working frequency channel in this paper is at the kHz level, and $f_{c}$ is set at 100 kHz in this system. The coils in the micro-SIM card and nano-SIM card are not the same size, and the values of $R_{1}$ and $C_{1}$ in these two kinds of SIM card are also different. The best distance is obtained at $R_{1}$ = 100 Ω and $C_{1}$ = 47 pF for the micro sim card, and $R_{1}$ = 10 Ω and $C_{1}$ = 2.2 nF for the nano-SIM card.

### 4.2. Rail-to-Rail Amplifiers

According to Equation (1), for example, the reader coil is 60 mm by 60 mm with 88 turns, and the coil in the nano SIM card is 11.8 mm by 8.8 mm with 18 turns. The voltage peak-to-peak value of the reader is 3.8 V, and the impedance of the reader coil is 56 ohm. $V_{s}$ = 50 μV at *x* = 8 cm, and $V_{s}$ = 20 μV at *x* = 12 cm according to Equation (1) in [20]. The max *x* is obtained when all amplifiers are at max gain, the total gain is 96,000, and the maximum value of *x* in theory is 16 cm; however, the actual maximum value *x* is 12 cm in [20], which means there are signal losses in analog frontend circuits.

In the RCC, the slave device should obtain the status whether it is in or out of the magnetic field. When $V_{s}^{\prime }$ is amplified by three amplifiers, six DACs and six comparators are used to detect magnetic field intensity. The six DACs are programed with different values, and the six comparators can obtain six signals with different period windows of value “1”, thus the action of in or out of magnetic field can be caculated by comparing the six period windows. A buffer is needed to increase signal drive capability because it will send it to six comparators. In this paper, we do not use the method involving the analog circuits of six DACs and six comparators. We use digital logic to detect the in or out of magnetic-field action. The LF reader sends several continuous preamble frame at the start of RCC communication and during the idle time. We can obtain the in or out status by recording whether it receives preamble frame at times.

The induced signal at the receiver coil is small, and the cascade amplifiers are used to produce the expected ideal waveform $V_{SIM}$; this process has no requirement in terms of gain accuracy. Three similar amplifiers are used with programmable gain in [20], as shown in Figure 3a. R7, C7, and R8, C8 are two low-pass filters. C10, C11, and C12 are blocking capacitors ($cap_{bl}$) which are used to add common voltage $V_{REF}$. The blocking capacitors are large (at the pF level) and suffer mismatch issues. These filters and blocking capacitors increase the signal loss in the amplify process.

**Figure 5:**
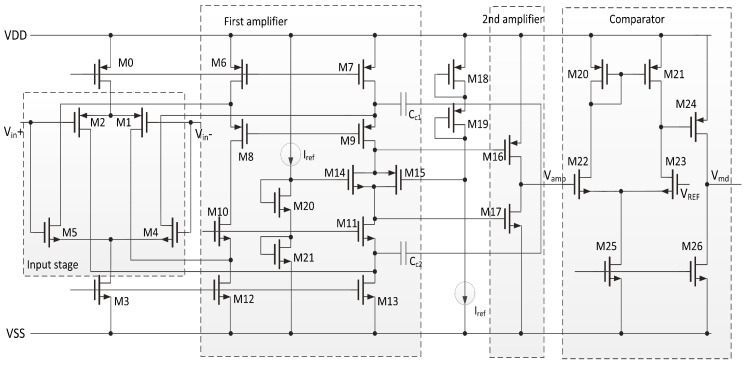
Cascade amplifier circuit.

In this paper, we simplify the design of analog frontend circuits as shown in Figure 3b. Two-stage rail-to-rail amplifiers with fixed gain of 51,200 are used instead of three programmable gain amplifiers, as shown in Figure 5. Because there is a LPF in coil matching circuit in Figure 4, there is no extra LPF in proposed rail-to-rail amplifiers, nor are the large blocking capacitors needed. In Figure 5, M1, M4 and M2, M5 are rail to rail input stage. M8, M9, M10, M11, M14 and M15 are first-stage amplifier. M16 and M17 are second-stage amplifier [24,25], and these two transistors also form a class AB amplifier. Table 1 gives the transistor dimensions of the two amplifiers. For example, M0 consists of 8 transistors with a width of 12 μm and a length of 4 μm. The simulation shows the phase margin of rail-to-rail amplifiers is larger than 52°.

The LF communication distance *x* is only a few cm. Considering that different mobile phones have different magnetic field shielding strengths, and the LF reader coil is installed some distance from the surface of the master device, *x* needs to be improved as much as possible to allow space for installment and to improve the application convenience of the RCC. In place of *x*, $V_{amp}$ is similar to the ideal waveform $V_{SIM}$. For x+i, $V_{amp}$ is worse than $V_{SIM}$, and a comparator is used to produce the ideal waveform $V_{SIM}$. The comparator is used to compare the $V_{REF}$ and $V_{amp}$ and to output “0” or “1”.

### 4.3. Self-Correcting Clock Recovery Differential Manchester Decoder

There are many methods of differential Manchester decoding. The basic method is based on the accurate clock counter to identify “1” and “0” [26]. This method requires high chip-clock accuracy and consistency, and the period of the LF working frequency $T_{m}$ being fixed at the transmitter and receiver. In CMOS technology, the clock generated by the oscillator using a resistive and capacitive network (RCOSC) is different from that in chips. The clock frequency deviation can range from −50 to +50% at the worst if the oscillator is not trimmed.

In previous work [20], a high-level threshold register and a low level threshold register were used to generate a decoding clock, and this design can adjust to a clock frequency deviation of ±30%. However, this method needs a high system clock to get a large value of high and low-level threshold register. Meanwhile, when the working frequency of LF is changed, a new value of high- and low-level threshold registers should be reloaded by the SE chip.

**Figure 6:**
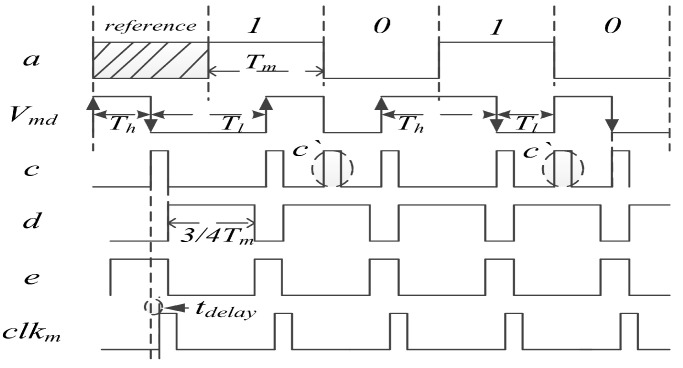
Decoding process of differential Manchester code.

In this paper, a self-recovery clock method is used to eliminate the dependence on system clock precision and adjusted to different LF working frequencies, as shown in Figure 6. In the differential Manchester code, a clock is generated at the fall and rise edge of $V_{md}$. In the generated clocks, there are some redundant clocks, so a delay time is used to mask the redundant clocks, and what are left arethe recovery decoding clocks. The process is as follow: First, two consecutive high-level and low-level periods are counted as $T_{h}$ and $T_{l}$. When $T_{h}$:$T_{l}$ is ≈ 1:2 or 2:1, $T_{m}$ can be obtained by $T_{m}$ = $max(T_{h},T_{l})$, this happens when the differential Manchester code is changing (1 → 0 or 0 → 1). Signal *c* is obtained by detecting the rising and falling edges of $V_{md}$. Signal *d* is obtained by broadening the pulse period of *c* to 3/4 $T_{m}$. Using inverted *d* to mask the redundant pulse $c^{‘}$ in *c*, the recovery clock $clk_{m}$ is obtained, and the decoded message *a* can be obtained by sampling $V_{md}$ using $clk_{m}$. Compared with the original clock counter method, this method can adapt to a variety of signal periods.

### 4.4. Low Power Design

**Figure 7:**
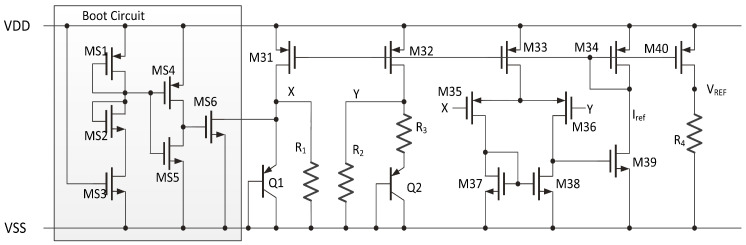
Bandgap circuit.

The proposed analog frontend circuits are simple compared to those in [20]. The other circuits are auxiliary circuits including a bandgap (BG), two voltage regulators (VR), and a RCOSC. The BG provides the reference voltage $V_{REF}$ and current $I_{ref}$ for the analog frontend circuits. Figure 7 gives the circuits of bandgap [27]. $V_{REF}$ = 1V in this work. MS1, MS2, MS3, MS4, MS5 and MS6 are boot circuits for bandgap. Table 2 gives the transistor dimensions of the banggap circuits. One VR is for the analog frontend circuits which is 2 V, and the other one is for the digital logic and is 1.8 V. The SPI interface is designed to be asynchronous to avoid the need for a high working frequency clock. This means that the clock signal of SPI interface is used directly. The primary threats to asynchronous circuits are glitches in the SPI interface pins. We add a resistance-capacitance (RC) passive filter and a Schmidt trigger with hysteresis comparison in input-output (IO) circuits to remove glitches. Because the proposed differential Manchester decoding method uses a self-recovery clock and is asynchronous to system clock, the system clock has no accuracy or high-frequency requirements. A low frequency 50 KHz RCOSC can be used without trimming. The RCOSC is designed in low power mode that works in the subthreshold region with a current consumption of less than 500 nA [28]. The dynamic power of digital logic can be calculated as $P_{d}$ = $CV^{2}f$ [29]. Because the RCOSC frequency is low, i.e., 50 KHz compared to 360 KHz in [20], the power consumption of digital logic also decreases.

## 5. Range-Controlled Communication Protocols

The HF communication distance is at the meter level, and the LF communication distance is at the centimeter level. The RCC protocol restricts HF to work only when LF establishes a connection.

### 5.1. Frame Format of Low-Frequency Communication

**Figure 8:**
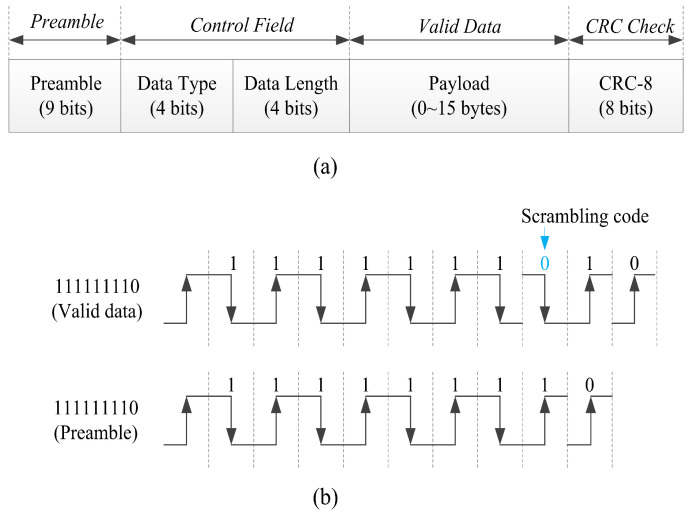
Frame format of LF transmission:(**a**) frame format. (**b**) scrambling code.

The frame format of LF is composed of the preamble, control domain, valid data, and cyclic redundancy check (CRC) bits, as shown in Figure 8a. The preamble is used for frame synchronization and consists of eight “1” bits and one “0” bit to form “111111110”, this is helpful to obtain $T_{m}$ for the proposed differential Manchester decoder. The form of preamble also means that eight continuous “1” bits cannot exist in next frame data to ensure the preamble form is unique. The scrambling mechanism is added at the LF reader to avoid eight consecutive “1” bits in the sequence. A scrambling code “0” is added after every seven consecutive “1” bits in the data stream as shown in Figure 8b. The LF receiver automatically processes the scrambling code to obtain the transmission data. The control domain defines the frame type (data frame or command frame) and data length. CRC is used to verify the control domain and valid data. The generated polynomial of CRC is $x^{8}+x^{2}+x+1$.

### 5.2. Protocol for Range-Controlled Communication

**Figure 9:**
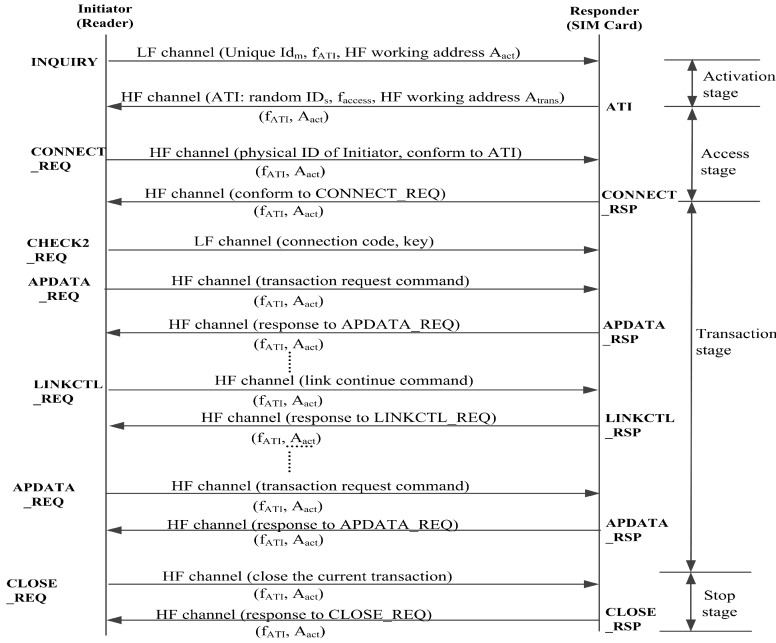
Protocols of proposed short-range communication.

Data transmission in LF is encrypted. The encryption process is carried out in the MCU of the master device, and decryption is carried out by the SE chip [30]. RCC must be carried out within the effective distance of LF, as described in Figure 9. The workflow of the proposed protocol session includes four stages: activation, access, transaction, and stop.

In the activation phase, the initiator first sends 20 preambles, then generates ${\mathrm{ID}}_{m}$, and calculates the HF activation response (ATI) frequency channel $f_{\mathrm{ATI}}$ according to ${\mathrm{ID}}_{m}$. If the HF frequency channel is currently occupied, ${\mathrm{ID}}_{m}$ needs to be regenerated until the selected ATI frequency channel is idle. The initiator sends the INQUIRY command with ${\mathrm{ID}}_{m}$ and $f_{\mathrm{ATI}}$ through the LF channel. The responder is activated after receiving the INQUIRY command and generates ${\mathrm{ID}}_{s}$ and the HF frequency channel $f_{\mathrm{access}}$ for the access and transaction stages. The responder also must ensure that the generated $f_{\mathrm{access}}$ is currently idle. The responder sends an ATI message, including ${\mathrm{ID}}_{s}$ and $f_{\mathrm{access}}$ through the HF channel at the working frequency $f_{\mathrm{ATI}}$. The responder enters the access phase after sending the ATI. If initiator doesnot receive the ATI message, it goes back to activation phase.

The HF channel in the access and transaction stages operates at frequency $f_{\mathrm{access}}$. The initiator sends a connection request CONNECT_REQ through the HF channel in the access phase. The responder receives the CONNECT_REQ command and sends the response CONNECT_RSP to the initiator within 8 ms. The responder enters the transaction stage after sending CONNECT_RSP.

The initiator sends an encrypted data exchange request APDATA_REQ through the HF channel in the transaction stage. At the same time, the LF channel is used to send the corresponding encryption key. Once the responder receives APDATA_REQ and the encryption key, it parses and executes the APDU command encapsulated in APDATA_REQ, then encapsulates the response in APDATA_RSP and sends it to the initiator. If the responder receives the wrong APDATA_REQ or the receiving time is beyond 100 ms, it will return to the activation stage. The responder will send APDATA_RSP or the long time wait frame (LTW) to the initiator within 500 ms and remain in the transaction stage. To maintain the transaction phase, the initiator sends the LINKCTL_REQ frame through the HF channel every 44 ms during the idle time of the transaction phase.

When the transaction stops normally or the responder’s status is abnormal, the initiator must send a CLOSE_REQ command to close the connection through the HF channel. After receiving the first correct CLOSE_REQ command, the responder immediately closes the transaction and returns to the activation stage.

## 6. Experiments

### 6.1. Setup

**Figure 10:**
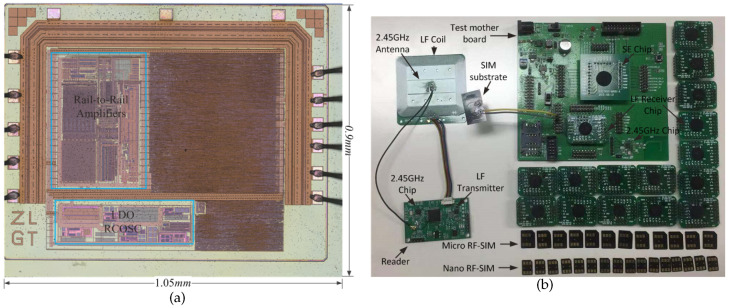
(**a**) Micrograph of the LF receiver. (**b**) Measurement environment of the proposed RCC system.

The LF receiver chip was fabricated using a 0.18 μm technology platform, as shown in Figure 10a. The die area is 1.05 mm × 0.9 mm, and the current consumption is 41 μA. The measurement environment of RCC is illustrated in Figure 10b. HF is a 2.45 GHz chip. The RCC reader board has a separate antenna printed circuit board (PCB) with an LF coil and an HF antenna, and it is usually installed on the surface of point-of-sales (POS) equipment. A USB2UART cable is used to connect the RCC reader board and computer and print RCC information on the computer. A development test board with the LF receiver chip, SE chip and HF chip is used to measure the waveform of the LF chip in air. Some LF receiver chips are packaged in chip-on-board mode (COB), and a empty flexible substrate of the SIM card is used because the LF receiver coil and coil matching circuits are inside it.

**Figure 11:**
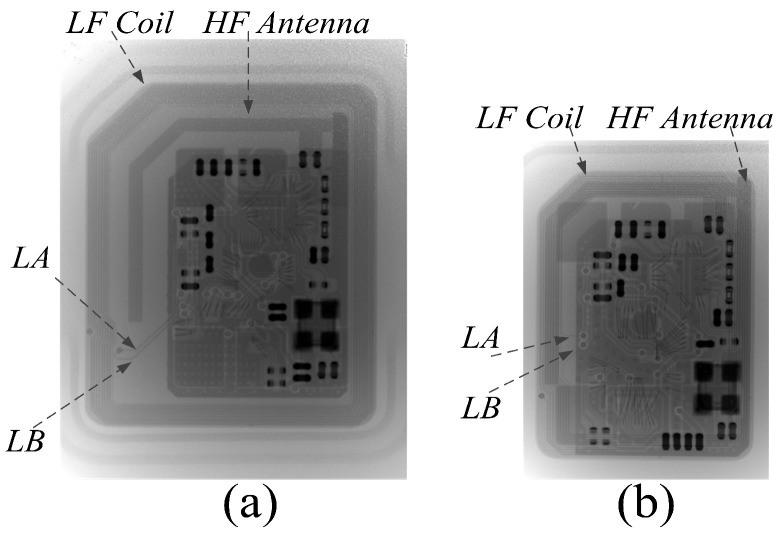
X-ray of SIM cards. (**a**) Micro-card. (**b**) Nano-card.

We also fabricated SIM cards with the SE chip, HF chip, and LF receiver chip. Figure 11 presents an X-ray graph of the SIM cards. The substrate of SIM card is double-sided PCB. The micro-SIM card has a large card size: the LF coil is outside, and the HF antenna is inside. The nano-SIM card is small: the LF coil and HF antenna are superimposed, the LF coil is on the bottom side and the HF antenna is on the top side. Because the die size is smaller than that of an SE chip, the SE chip and LF receiver have a package-in-package (PIP) design in both types of SIM card, and $N_{r}$ is increased four turns compared to that presented in [20]. The coil matching circuit is still a LPF, as shown in Figure 4.

### 6.2. Waveform of LF Analog Frontend Circuits

**Figure 12:**
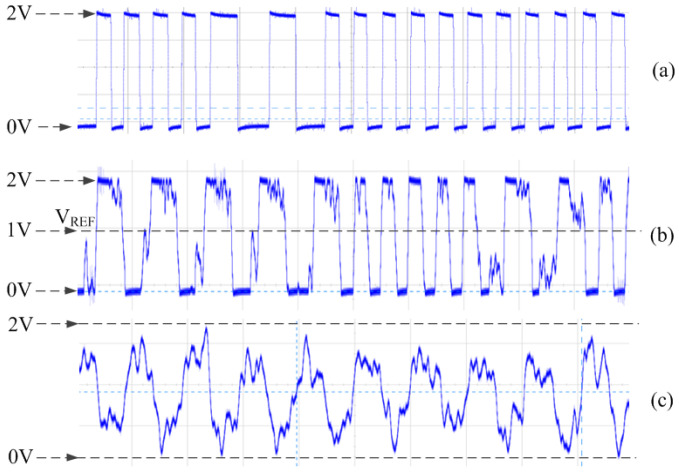
Waveform of $V_{amp}$ at *x*. (**a**) *x* < 11.5 cm. (**b**) *x* = 12 cm. (**c**) *x* = 13 cm.

Figure 12 shows the output voltage of $V_{amp}$ at 25 °C in air by COB at 4 KHz. The maximum communication distance is 12.3 cm. When the *x* distance is shorter than 11.5 cm, $V_{amp}$ is very close to the ideal waveform $V_{SIM}$, as shown in Figure 12a, and it can be directly sent to digital differential Manchester decoder. When *x*∈ (11.5 cm, 12.2 cm), $V_{amp}$ becomes worse, as shown in Figure 12b, and a comparator with judge threshold of $V_{REF}$ is used to obtain the digital signal $V_{md}$, which can still be decoded correctly. When *x* > 12.2 cm, the waveform of $V_{amp}$ is even worse, as shown in Figure 12c, thus, the qualified digital $V_{md}$ is too bad to be decoded, and LF communication fails.

We perform the LF data transmission experiments in the air by COB at 4 KHz like these: the LF reader sends a frame of 16 byte data 1000 times. If the LF receiver chip receives a frame, and the CRC check is correct, a data correct interrupt signal will be sent out and SE will read the data through SPI interface. $N_{se}$ is the number of times that SE has read the correct data which is equal to LF reader sends. We calculate the data transmission error rate as $p_{e}$ = (1000−$N_{se}$)/1000. The results show that $p_{e}$ = 0 when *x* < 11.5 cm. When *x*∈ (11.5 cm, 12.2 cm), $p_{e}$∈ (10%, 60%), and $p_{e}$ = 100% when *x* > 12.2 cm. In previous work [20], we find that when LF reader sends a frame with continuous “1”, *x* will decrease. The worst situation was occured when the LF reader sends a frame of 16 byte “FF”, *x* decreases as much as 2.5 cm. Since we use the new rail-to-rail amplifiers, we do not observe the distance decreases in data transmission experiments.

### 6.3. Comparison with Previous Work

In Table 3, we present a comparison of LF receiver chips. Compared to previous work, we do not use the LPF and large blocking capacitors ($cap_{bl}$) to avoid signal loss, and two-stage amplifiers are used instead of the previous three-stage amplifiers. The gain is 51,200 which can get the same communication distance as previous work. Besides, there is only one comparator (Comp) compared to six DACs and six comparators, and the OSC frequency is decreased from 360 KHz to 50 KHz. The proposed LF receiver chip proposed in this paper has a smaller die size and current consumption.

In [20], the development board was placed in different temperatures to study the effects on the LF receiver. We performed the same experiments, and Table 4 shows the comparison results. $COB_{ori}$ is the COB in [20] with two Π coil matching circuits. $COB_{ant}$ is the COB with the LF receiver in [20], and an improved LPF coil matching circuit in Figure 4. $COB_{pro}$ is the COB with the LF receiver proposed in this paper and an LPF coil matching circuit. $COB_{ant}$ achieves an almost 0.3 cm distance improvement compared to $COB_{ori}$. $COB_{pro}$ achieves the same *x* as $COB_{ant}$, and because $COB_{pro}$ has no current leakage at low temperatures, the distance decrease in low temperature for $COB_{ori}$ does not occur in $COB_{pro}$.

The proposed range-controlled communication system can adjust to different LF frequencies varying from 2 kHz to 4 kHz. The RCC has nearly 80 interactive communications in the transaction phase for one payment operation. The previous research have found that the larger the *x*, the larger the successful communication ratio. We compared the communication distance for the SIM card at 25 °C in different mobile phones of different companies for 2 kHz and 4 kHz. The test results are summarized in Table 5. $SIM_{ori}$ is the SIM card in [20], and $SIM_{pro}$ is the SIM card with the LF receiver proposed in this paper. The proposed SIM card has more than a 3 cm improvement as compared with previous work, and it still has 4.2 cm in phones with a metal shell, as VIVO V3M and Huawei Mate 9. The results also show that in the metal phones, the SIM card has a better distance at 2 KHz than 4 KHz, and the other phones behave better at 4 KHz than 2 KHz. The reason for this may be that the 2 KHz frequency has a better signal penetration than 4 KHz, and 4 KHz has lager magnetic-field intensity than 2 KHz. It is better to change LF frequency to obtain a better *x* for phones. In the activation phase, when it fails, the initiator changes the LF frequency, and re-performs the activation operation.

**Figure 13:**
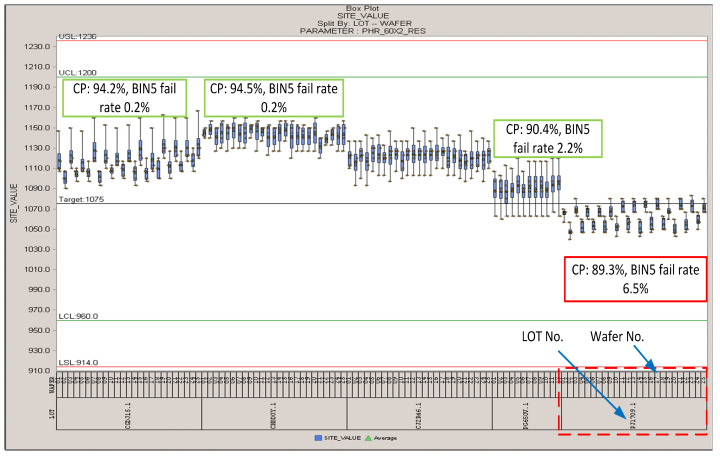
Variation of PHR_60X2_RES.

Many wafers are produced to support the RCC application. These wafers are given a circuit probing (CP) test before being packaged in the SIM card. The CP test vector collects the output of LDO and low power RCOSC. The foundry monitor the process variation in the manufacture process, and they found that one piece of wafer’s PHR_60X2_RES window is lower than the target value of 1075, as shown in Figure 13. THE CP test found that the output frequency of RCOSC is lower to −50% in this wafer. We packaged 200 SIM cards using the LF receiver chip in this wafer, and re-performed the experiment in Table 5 to study the effect of this situation. The results are the same as in Table 5. This means that in the proposed RCC methods, the frequency of RCOSC has little effect on communication distance *x*.

Some smartphones use more than two SIM cards on one card seat. The inner SIM card is placed in the middle of the mobile phone, and the HF signal is poorly disturbed by the device, with the situation worsening if the phone made with a completely metallic shell. In this case, we found that the RCC distance is determined by HF. In the activation phase, the LF reader synchronously transmits the transmission power parameter of HF. If the HF response is not received or the received signal strength indication (RSSI) is too low, the initiator returns to the activation phase and increases the transmission power parameter of HF to ensure that the communication distance is completely determined by LF.

## 7. Conclusions

In this paper, we present a range-controlled communication system using an improved LF communication system. The circuit of the LF transmitter comprises class-D amplifiers to obtain a large magnetic field. The LF receiver consists of rail-to-rail amplifiers and only one comparator. The LF receiver chip is fabricated using a 0.18 μm CMOS technical platform with a die area of 1.05 mm × 0.9 mm and current consumption of 41 μA. The differential Manchester decoding circuit in the LF receiver is designed as a self-recovery clock recovery circuit to adopt different LF frequencies, such as 2 kHz and 4 kHz. The proposed protocol restricts the HF communication distance by using LF to transmit the working frequency and communication key of the HF channel.

## Figures and Tables

**Table 1:** Transistor dimensions of the two amplifiers.

MOS No.	Dimension (μm/μm)	MOS No.	Dimension (μm/μm)	MOS No.	Dimension (μm/μm)
M0	(12/4) × 8	M1	(8/4) × 7	M2	(12/4) × 12
M3	(4/4) × 8	M4	(12/4) × 4	M5	(12/4) × 4
M6	(14/4) × 8	M7	(14/4) × 8	M8	(12/4) × 8
M9	(12/4) × 8	M10	(8/4) × 4	M11	(8/4) × 4
M12	(8/4) × 7	M13	(8/4) × 7	M14	(8/2) × 1
M15	(8/2) × 1	M16	(8/2) × 4	M17	(8/2) × 4
M18	(8/2) × 1	M19	(8/2) × 1	M20	(8/2) × 1
M21	(8/2) × 1	$C_{c1}$	0.5 pF	$C_{c2}$	0.5 pF

**Table 2:** Transistor dimensions of bandgap circuits.

MOS No.	Dimension (μm/μm)	MOS No.	Dimension (μm/μm)	MOS No.	Dimension (μm/μm)
MS1	(2/1) × 1	MS2	(1/40) × 1	MS3	(2/1) × 1
MS4	(2/0.5) × 1	MS5	(2/0.5) × 1	MS6	(2/1) × 1
M31	(3/5) × 2	M32	(3/5) × 2	M33	(3/5) × 1
M34	(3/5) × 1	M35	(10/8) × 2	M36	(10/8) × 2
M37	(2/10) × 1	M38	(2/10) × 1	M39	(2/10) × 1
M40	(3/5) × 2	$R_{1}$	1.26 MΩ	$R_{2}$	1.26 MΩ
$R_{3}$	150.6 kΩ	$R_{4}$	898.4 kΩ	-	-

**Table 3:** Comparison of LF receiver chip.

Item	DIE Size (mm × mm)	Current	Gain	LPF	${\mathrm{cap}}_{\mathrm{bl}}$	Amplifiers	DAC	Comp	OSC
[20]	1.85 × 1.65	330 μA	96,000	2	3	three-stage	6	6	360 KHz
Proposed	1.05 × 0.9	41 μA	51,200	0	0	two-stage	0	1	50 KHz

**Table 4:** RCC Distance at Different Temperatures in Air for 4 kHz.

Distance (cm)	−40 °C	−20 °C	0 °C	25 °C	60 °C	85 °C
$COB_{ori}$	11.2	11.8	12.0	12.0	12.0	12.0
$COB_{ant}$	11.5	12.2	12.3	12.3	12.3	12.2
$COB_{pro}$	12.2	12.3	12.2	12.3	12.2	12.2

**Table 5:** Short-Range Communication Distance for Different Mobile Phones.

Items	Iphone 6	Iphone 6Plus	VIVO V3M	Huawei Mate 9	Iphone 11
Distance (cm, 2 kHz, $SIM_{ori}$)	6.8	3.0	1.2	1.0	4.3
Distance (cm, 4 kHz, $SIM_{ori}$)	7.8	2.1	<0.5	<0.5	5.6
Distance (cm, 2 kHz, $SIM_{pro}$)	8.8	5.5	4.2	4.1	6.5
Distance (cm, 4 kHz, $SIM_{pro}$)	9.7	4.6	3.1	2.9	7.7
